# Technical Report: Protocol for Characterizing Phenotype Variants Using Phenome-Wide Association Study (PheWAS) Utilizing the Nationwide Inpatient Sample 2020 in Individuals With Pancreatic Cysts and Lung Cancer

**DOI:** 10.7759/cureus.50982

**Published:** 2023-12-23

**Authors:** Samuel Y Huang, Reyes Johnathan, Neal Shah, Pranay Srivastava, Alexander A Huang, Frank Gress

**Affiliations:** 1 Internal Medicine, Icahn School of Medicine at Mount Sinai South Nassau, Oceanside, USA; 2 Gastroenterology, Icahn School of Medicine at Mount Sinai, New York, USA; 3 General Surgery, Northwestern University Feinberg School of Medicine, Chicago, USA

**Keywords:** icd-10-cm guidelines, statistical methodology, manhattan plot, lung cancer, pancreatic cysts

## Abstract

This technical report serves as a comprehensive guide for conducting a phenome-wide association study (PheWAS) utilizing data extracted from the Nationwide Inpatient Sample 2020. Specifically tailored to individuals diagnosed with pancreatic cysts and lung cancer, the report establishes a step-by-step workflow designed to assist researchers in uncovering potential associations within this specific cohort. The methodology outlined in the report ensures clarity and reproducibility by employing a curated cohort sourced from the GitHub repository and executed using R for robust data analysis. The code encompasses pivotal steps, including the utilization of a QQ plot as a crucial diagnostic tool aimed at identifying systematic biases or associations. Additionally, the report incorporates the creation of a Manhattan plot, delving into essential mathematical considerations to enhance the interpretability of the results. Notably, the report elucidates the handling of the International Classification of Disease version 10 (ICD-10) codes, providing a sample approach for their segmentation to analyze associations by diagnostic categories. The segmentation aligns with the guidelines outlined in the American Medical Association's ICD-10-CM 2022, the Complete Official Codebook with Guidelines (American Medical Association Press, 2021), ensuring a standardized and rigorous analytical process. This comprehensive guide equips researchers with the tools and insights needed to navigate the complexities of PheWAS within the context of pancreatic cysts and lung cancer, fostering transparency, reproducibility, and meaningful scientific exploration.

## Introduction

Phenome-wide association studies (PheWAS) represent a powerful approach in the realm of multi-phenotype analyses, offering a unique avenue to explore associations between genetic variants and a myriad of phenotypes within a given population [1]. The distinctive strength of PheWAS lies in its capability to associate not only well-known or prevalent phenotypes but also unearth connections with previously unknown or underappreciated health indicators [2,3]. This multifaceted strategy serves to unravel intricate disease mechanisms by mediating phenotypes and can potentially enable the identification of predictive biomarker phenotypes for specific health outcomes [4,5].

The significance of conducting PheWAS on electronic health records (EHRs) becomes particularly pronounced in the contemporary healthcare landscape. EHRs encapsulate a wealth of information, including unstructured clinical notes, laboratory test results (captured through Logical Observation Identifiers Names and Codes (LOINC)), billing codes such as the International Classification of Disease (ICD) and Current Procedural Terminology (CPT), as well as pharmaceutical data denoted by prescription information (RxNorm) [6]. The widespread adoption of EHR technology in the United States from 2008 to 2015 has resulted in a comprehensive repository of patient health information, making EHRs an invaluable resource for research and analysis [1]. The widespread adoption of electronic medical record (EMR) systems in routine clinical practice has transformed patient care, reducing costs and providing a longitudinal record of care for clinical and translational research. These systems house diverse data sources, including billing information, laboratory results, medication records, and clinical documentation, proving invaluable for clinical research studies and focused genomic investigations. The USA's prevalent use of the ICD, version 10, codes in clinical encounters, totaling over 14,000 disease codes in a hierarchical structure, provides an extensive framework for billing and research purposes. This structured data, combined with natural language processing algorithms, enables the extraction of information from unstructured clinical documentation, enhancing the understanding of individual phenotypes.

However, the raw nature of EHR data introduces challenges, as they represent an indirect reflection of the true patient state due to the intricacies of the recording process. Patient states exhibit variability over time, influencing the value, presence, type, and timing of recorded measurements [6,7]. As EHR data becomes increasingly comprehensive, there is a pressing need for sophisticated, integrative machine learning approaches to effectively model and derive meaningful insights from this vast and dynamic healthcare dataset. Additionally, despite the concept of a phenome-wide scan existing in the literature, many physician scientists still do not have widespread access to such computational capabilities. A translatable, systematic, high-throughput, and reproducible methodology for such has been lacking in the era of increasing healthcare data. In this article, the authors outline an applicable interactive algorithm designed for an initial PheWAS. This algorithm utilizes readily available ICD-10 codes, offering a means to replicate known phenotype-comorbidity associations and suggest novel potential associations.

This study aims to navigate these complexities and harness the potential of PheWAS on EHRs, shedding light on novel associations and contributing to the evolving landscape of precision medicine. The study utilizes a curated dataset from the Nationwide Inpatient Sample 2020, a national inpatient database that comprises information in ICD-10 codes, to create an interactive process on which healthcare practitioners can base their research and start generating hypotheses.

## Technical report

The overarching goal of the technical code is to introduce an interactive code that can be applied to preprocessed data for the purpose of executing PheWAS. The approach to dataset creation encompasses key steps, beginning with population selection, identification of relevant ICD-10 codes, comprehensive prevalence calculation, and multivariate regression analysis to quantify the odds of specific health outcomes. The case below involves utilizing the Nationwide Inpatient Sample 2020 and identifying ICD-10 codes for the cohort that had pancreatic cysts. The identification of ICD-10 codes focused on pancreatic cysts and lung cancer involved a systematic and collaborative approach led by an expert panel of interventional endoscopists, gastrointestinal fellows, and medical residents. The criteria for selecting ICD-10 codes were grounded in the American Medical Association's ICD-10-CM 2022 codebook and consensus decision-making within the panel. Demographic data was calculated and multivariate regression was used. This data can be found below:

The technical report assumes a basic understanding of R programming, which can be downloaded from the R Project website at https://www.r-project.org/ and from the nearest CRAN mirror location.

Reading in the data

The dataset can be downloaded from the curated GitHub repository at https://github.com/huangs8/PancreaticCystsandLungCancer

The initial steps involve reading in the data, running the QQ plot, and implementing the PheWAS followed by cleaning up and grouping ICD-10 codes according to desired clustering. First, read in the data and the necessary packages.

manhattan_data = read_csv("pancreaticcystlungcancer.csv")

# Install and load necessary packages

library(dplyr)

library(ggplot2)

library(ggrepel)

library(qqman)

Quantile-quantile plot

The quantile-quantile plot (QQ plot) is a graphical tool used to assess whether the distribution of observed p-values in a dataset deviates from the expected distribution under the assumption of no association (null hypothesis). In the context of genetic association studies, such as a PheWAS, the QQ plot is particularly valuable.

**Figure 1:**
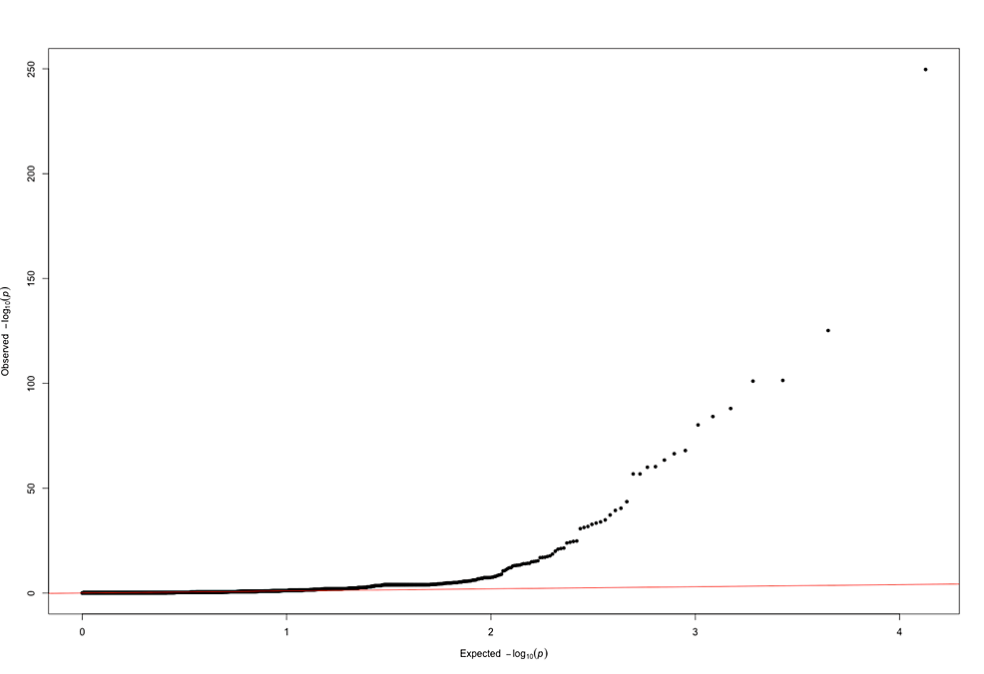
QQ plot QQ plot illustrating p-values for the association between each ICD-10 code and the odds of pulmonary cancer

The significance of the QQ plot lies in its ability to visually identify deviations from the expected uniform distribution of p-values. If the observed p-values follow the expected distribution, the points on the QQ plot will fall along a straight line. Deviations, however, may indicate potential issues such as systematic biases, population stratification, or other confounding factors. Figure 1 below is the QQ plot for the PheWAS values.

# Create a QQ plot

qq(manhattan_data$P, main = "QQ Plot for PheWAS Values")

Manhattan plot

**Figure 2:**
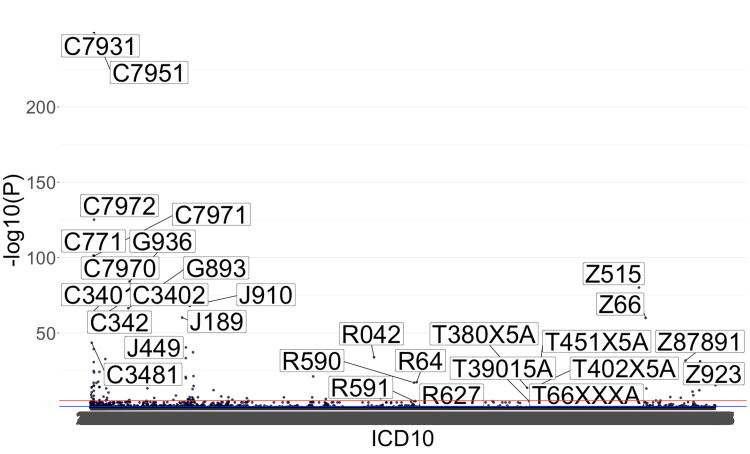
PheWAS of the Nationwide Inpatient Sample 2020 in individuals with pancreatic cysts and lung cancer The figure depicts a Manhattan plot illustrating the results of a PheWAS. The x-axis represents the International Classification of Disease version 10 (ICD-10) codes without abbreviations. On the y-axis, the inverse log p-value derived from a multivariate model predicting lung cancer in patients with pancreatic cysts showcases the statistical significance of associations. Each data point corresponds to a particular ICD-10 code and its degree of significance

In a PheWAS, the Manhattan plot serves as a visual representation of the statistical significance of associations between genetic variants and a multitude of phenotypes. The process involves extracting p-values from the statistical analysis, which quantifies the strength of the relationship between genetic markers and specific health outcomes. The Manhattan plot derives its name from the distinctive skyline-like appearance created by plotting the negative logarithm (base 10) of the p-values on the y-axis against the genomic position of each variant on the x-axis. This transformation enhances the visibility of significant associations, as they manifest as peaks reaching higher on the plot. Thus, a higher peak indicates a more statistically significant association. Figure 2 shows the PheWAS as generated by the interactive code below.

# Filter out specified ICD10 codes to remove

manhattan_data <- manhattan_data %>%

 filter(!SNP %in% snps_to_remove)

 

# Prepare the dataset

don <- manhattan_data %>%

 

 # Compute How many clusters of ICD10 codes to be grouped

 group_by(CHR) %>%

 summarise(chr_len = max(BP)) %>%

 

 # Calculate cumulative position of each ICD10 grouping

 mutate(tot = cumsum(chr_len) - chr_len) %>%

 select(-chr_len) %>%

 

 # Add this info to the initial dataset

 left_join(manhattan_data, ., by = c("CHR" = "CHR")) %>%

 

 # Add a cumulative position of each ICD10 code

 arrange(CHR, BP) %>%

 mutate(ICD10 = BP + tot) %>%

 

 # Add highlight and annotation information

 mutate(is_highlight = ifelse(SNP %in% snpsOfInterest, "yes", "no")) %>%

 mutate(is_annotate = ifelse(-log10(P) > 4, "yes", "no"))

 

# Prepare X axis

axisdf <- don %>% group_by(CHR) %>% summarize(center = (max(ICD10) + min(ICD10)) / 2 )

 

# Make the plot with title

ggplot(don, aes(x = ICD10, y = -log10(P))) +

 

 # Show all points

 geom_point(aes(color = as.factor(CHR)), alpha = 0.8, size = 1.3) +

 scale_color_manual(values = rep(c("grey", "skyblue"), length(unique(don$CHR)))) +

 

 # custom X axis:

 scale_x_continuous(label = axisdf$CHR, breaks = axisdf$center) +

 scale_y_continuous(expand = c(0, 0)) + # remove space between plot area and x axis

 

 # Add highlighted points

 geom_point(data = subset(don, is_highlight == "yes"), color = "orange", size = 2) +

 

 # Add label using ggrepel to avoid overlapping

 geom_label_repel(

 data = subset(don, is_annotate == "yes"),

 aes(label = SNP),

 size = 2,

 max.overlaps = 10 # Set max.overlaps to Inf to allow all labels

 ) +

 

 # Custom the theme:

 theme_bw() +

 theme(

 legend.position = "none",

 panel.border = element_blank(),

 panel.grid.major.x = element_blank(),

 panel.grid.minor.x = element_blank()

 ) +

 

 # Add title

 ggtitle("Phenome Wide Association Study of the Nationwide Inpatient Sample 2020 in Individuals with Pancreatic Cysts and Lung Cancer")

This large-scale application is meticulously crafted for healthcare clinicians, providing them with a user-friendly interface to harness the power of the PheWAS paradigm within EMRs. Representing an unbiased approach to both replication and discovery, this innovative tool delves into the intricate relationships between targeted diagnoses and a wide array of phenotypes. Its foundation is rooted in the rich landscape of EHR data, particularly involving ICD-10 codes.

The Bonferroni correction addresses the issue of multiple testing in the analysis of PheWAS results. The stringent Bonferroni correction method adjusts the significance threshold for statistical tests to control the family-wise error rate. When we implemented this correction, only a few disease-gene associations remained statistically significant. This conservative correction method is known for its strictness, often leading to increased p-values required for significance. While the Bonferroni correction is a powerful tool to minimize false positives, it can be overly conservative in the context of PheWAS due to the high dimensionality of the data and the relatively low prevalence of many diseases [8].

## Discussion

The above introduces a healthcare-friendly, interactive workflow that demonstrates the utility of PheWAS in the context of clinical reasoning and interventions. By leveraging the wealth of information embedded in ICD-10 codes within EHRs, the workflow presents a valuable pathway toward improving the diagnosis of common medical conditions.

**Figure 3:**
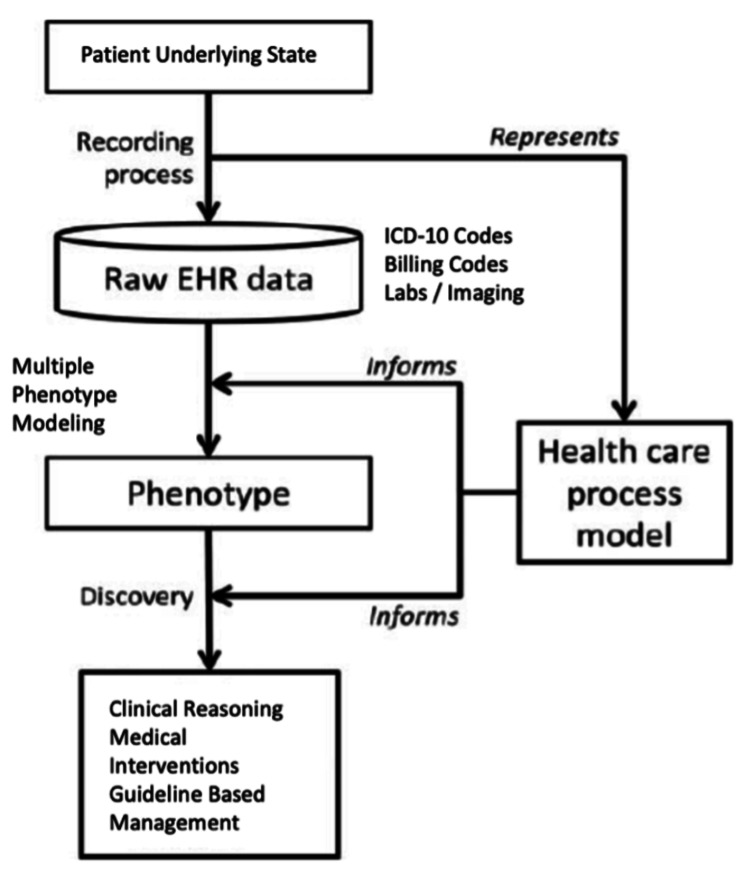
Schema for the integration of multiple phenotype models such as PheWAS in current studies EHR: electronic health record, ICD: International Classification of Diseases

Figure 3 illustrates the pivotal role of PheWAS modeling within the broader framework to improve clinical decision-making, medical interventions, and guideline-based management. The patient's underlying true state, indirectly measured through the recording process and raw EHR data, serves as the foundation for understanding disease phenotypes. Various aspects of a patient's history, such as pack years of smoking, X-ray reads, spirometry values, bloodwork, physical exams, and other relevant factors, are recorded in the EHR. By employing multiple phenotype modeling, such as PheWAS, associations between different phenotypes are brought to light, either validating or challenging existing assumptions. For instance, associations between lung cancer history and diverse indicators, including paraneoplastic syndromes, can be explored. This comprehensive approach enhances clinical reasoning, facilitates more informed medical interventions, and contributes to the refinement of guideline-based management strategies, ultimately advancing patient care.

It is crucial to acknowledge certain limitations inherent in utilizing raw EHR data [9,10]. The data, being an indirect reflection of the true patient state due to the recording process, introduces variability stemming from the dynamic nature of the patient's health status [11]. Patient states can significantly influence the value, existence, type, and timing of recorded measurements, thereby impacting the precision of analyses. Other limitations include the fact that many rare diseases have small sample sizes for individual disease codes, and as a result, the potential for enhanced power and cross-institutional application is recognized. However, these are becoming more and more realities as electronic healthcare becomes more and more centralized. Future refinement, incorporating laboratory data, natural language processing, and machine learning algorithms, may improve case accuracy [12].

Despite these limitations, our approach offers substantial benefits. The application of PheWAS, coupled with ICD-10 codes, enables the inference of pleiotropic variants and pathways implicated in multiple phenotypes [3]. PheWAS exhibits strength in its applicability to any EHR system and can be seamlessly updated to incorporate new ICD-10 classifications, ensuring its adaptability to evolving medical standards and enhancing its utility in precision medicine research. This includes the identification of shared risk variants, leveraging summary statistics-based methods, and elucidating distances based on effect size [13]. Additionally, the integration of phenotypic risk prediction facilitates the exploration of multiple phenotypes sharing at the pathway level [13]. These insights contribute to a more nuanced understanding of disease mechanisms, paving the way for enhanced clinical decision-making and targeted interventions.

## Conclusions

While recognizing the challenges associated with raw EHR data, the provider-friendly PheWAS workflow stands as a valuable tool for clinicians, offering a pathway to refine clinical reasoning and interventions through the effective utilization of ICD-10 codes. The benefits derived from the integration of genetic information underscore the potential of this approach for uncovering shared mechanisms underlying diverse phenotypes and advancing precision medicine.
